# Supplementary material for: After action review of the response to an outbreak of Lassa fever in Sierra Leone, 2019: Best practices and lessons learnt
Source: PLoS Negl Trop Dis. 2022 Oct 5;16(10):e0010755. doi: 10.1371/journal.pntd.0010755 (PMC9534430; doi:10.1371/journal.pntd.0010755)
Supplement: S1 File — (PDF) [file pntd.0010755.s001.pdf]

MINISTRY OF HEALTH AND SANITATION

THE REPUBLIC OF SIERRA LEONE

Learning from Incidents and Exercises

Guidance on EPRR Debriefing and After Action Reviews

"We learn from experience that men never learn anything from experience!"

George Bernard Shaw

Index

Abbreviations and Acronyms **3**

1 Introduction **4**
2 Methodology **5**
3 Process **6**
4 Building the After-Action Review Facilitation Team **7**
5 Preparing for an After-Action Review **9**
5.1 An Example of a coordination Pillar Trigger Question **10**
6 Conducting a Hot Debrief **11**
6.1 Draft Agenda Hot Debrief Template **12**
6.2 Key Facilitator Notes for Hot Debrief **13**
6.3 Template Hot Debrief Form **14**
6.4 Key Area Questions **15**
7 Conducting an After-Action Review **16**
7.1 Example of Structured Pillars **17**
7.2 Example of a half day Workshop Agenda **18**
7.3 Key note for After-Action Review Debrief Facilitator **19**
7.4 Template After-Action Review Debrief Form **20**
7.5 Example Trigger Questions **21**
7.5.1 Example Trigger Questions **22**
7.5.2 Example Trigger Questions **23**
7.5.3 Example Trigger Questions **24**
8 Example of an After-Action Review PowerPoint Slide Show **25**
9 Evaluation of IHR (2005) Core Capacities **31**
9.1 IHR Capacity and Indicators **31**
10 After-Action Review Report **33**
10.1 Links to the IHR MEF **33**
10.2 After-Action Review Report Template **34**
11 After-Action Review Action Plan **37**
11.1 Action Plan Template **37**
12 Annex A Definition of AAR Indicators Goal-based Evaluation Ratings **38**
13 Sources **39**

ABBREVIATIONS AND ACRONYMS

AAR	After Action Review
EPRRG	Emergency Preparedness Resilience and Response Group
GOARN	Global Outbreak Alert and Response Network
IGO	Intergovernmental Organisation
IHR	International Health Regulations
IHR MEF	International Health Regulations Monitoring & Evaluation Frame-work.
IM	Incident Manager
IMS	Incident Management System
IPC	Infection Prevention and Control
JOR	Joint Operational Review
MDA's	Ministries, Directorates and Authorities
M&E	Monitoring and Evaluation
MoHS	Ministry of Health and Sanitation
NAPHS	National Action Plan for Health Security
NGO	Non-Governmental Organisation
NIERP	National Incident and Emergency Response Plan
PHEIC	Public Health Event of International Concern
RRT	Rapid Response Team
SIMEX	Simulation Exercise
SOP	Standard Operating Procedure
SPAR	State Party Self-Assessment Annual Reporting
WASH	water and sanitation for health
WHO	World Health Organisation

1 Introduction

The importance of reflective practice has been identified for many years, but it has only been over the last few years that there has been a greater drive for change. Debriefing is now at the forefront of many key changes within policy and practice within the Ministry of Health and Sanitation (MoHS) and other Ministries, Departments and Agencies (MDA's), which is key to Joint Organisational Learning.

The MoHS advocates that a structured debrief should be considered after a response to any event with public health significance. Such debriefs following an incident/outbreak or simulation exercise indicates that debriefing is a planned activity, designed to initiate the process of identifying lessons and support the next steps towards learning them. To this end, the guidance provided in this document supports the Lessons Learned section within the Multi Hazard Public Health National Incident and Emergency Response Plan (NIERP) and the International Health Regulations (IHR 2005) Monitoring and Evaluation Framework (IHR MEF).

Additionally, an After Action Review (AAR), is one component of the IHR MEF. The Framework is comprised of four component areas detailing a mixed approach of qualitative and quantitative data collection and analysis. In addition, desk reviews are undertaken as well as functional assessments of capacities for prevention, preparedness, detection and response.

The four components are; the compulsory State Party self-assessment Annual Reporting (SPAR), and three voluntary components, External Evaluation, the AAR and the Simulation Exercises (SimEx).

Best practice suggests that it is important to review and assess any actions taken as part of a public health response, in order to capitalise on best practices, identify areas and actions for improvement, and promote individual and collective learning.

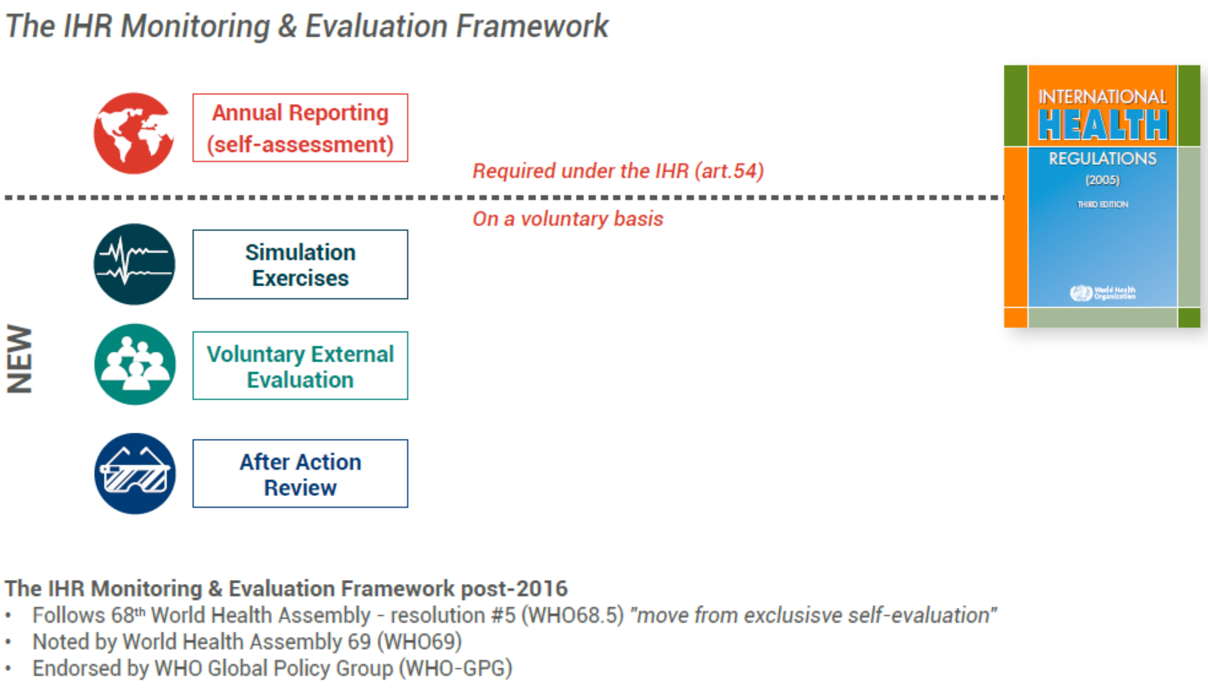

2 Methodology

There are several recognised methodologies for capturing observations and data from real incidents or exercises, with the main one being the post-event debrief, also known as an After Action Review. After Action Reviews were first used in the US Military to provide feedback on the success of a mission, whilst identifying any lessons to be learnt from a given activity.

With the evolution of time, many training institutions have adapted these different methodologies as a means of implementing a lesson identified process. This document provides a practical tool to assist in the evaluation of incidents/outbreaks and exercises, whilst facilitating the learning outcomes from these events.

As such, the type of debrief processes used by the MoHS are:

- **Hot Debrief -** this must be held immediately after the incident/outbreak/exercise or once a shift or response is completed and is often referred to as "hot wash up". It is important to capture the views of participants whilst they are fresh in individual minds
- **Internal Organisational Structured AAR** – ideally this should be held within 2 – 4 weeks of the incident/outbreak/exercise. Again, this is a structured process which allows participants some reflective time to consider their views.
- **Multi-Agency AAR** – ideally this should be held within 4 – 6 weeks of the incident/ outbreak/exercise and allows for participation of MDA's and Partners.

Within the MoHS, both Internal Organisational Structured AAR's and Multi-Agency AAR's will be undertaken by an independent facilitator appointed by the Director DHSE. In all cases, strict timelines for delivery will be adhered to, in support of the lessons learned process. Therefore, AAR's (AAR) should be undertaken within six weeks (maximum) of the end of an incident/outbreak or simulation exercise.

Debriefing is an essential part of the emergency planning cycle and is key to system development through experiential learning. Essentially, this is learning through first-hand observations and is used to support best practice and to inform system improvement and development.

In respect of simulation exercises, structured debriefs should be planned into the exercise design phase. This will ensure that feedback is focused and demonstrates a commitment by the MoHS to undertake reflective practices on all occasions.

The process of reviewing incidents, adopted by the MoHS, includes consideration of the routes by which further learning is disseminated once it has been identified. This responsibility sits ultimately with the National Public Health Emergency Management Committee (PHEMC) with some delegated authority to the Emergency Preparedness Resilience and Response Group (EPRRG) for lower level incidents.

The MoHS recognises that an AAR is not the "**cure-alls**" for Directorate's training problems. Leaders within each team must still take responsibility for training their staff and team members in the emergency response roles that they could undertake. However, AARs are a key part of the training process aimed at improving team performance.

3 Process

The lessons identified process is in line with the MoHS governance and M&E framework within the NIERP and its statutory duties under the IHR (2005). Any debrief will provide a *"***safe***"*, non-confrontational environment in which participants can identify important issues and associated learning. The debrief does not seek to identify ***all*** the issues associated with an incident. Instead it seeks to clarify key issues at the start of the learning process by:

- Outlining a common view of what happened
- Identify strengths to maintain and areas for improvement
- Consider why and what happened to support recommendations for improvement and developments can be made.

Taking an integrated approach, the debrief process will provide a forum for those involved in the real incident or exercise to express their views in a **blame free** environment. The MoHS accepts that mistakes will happen and that often they have many masters. Blame rarely lies in one place, so it is vital to stress from the outset that the debrief is **not about** apportioning blame.

It is anticipated that the guidance will produce a consistent level of quality by following an agreed process of best practice. Therefore, irrespective of the debrief process used, an incident/outbreak/exercise report will be published with identified recommendations and an Action Plan for implantation.

As detailed in the NIERP, the Incident Manager by default is responsible for ensuring that the lessons identified process takes place. The Incident Manager will decide who is to manage the debriefing process and agree with them the terms of reference for the debriefing. There are several AAR common objectives for consideration, which could include:

- ***Assessing the functional capacity of existing systems to prepare, prevent, detect and respond to a public health event***

- ***Identifying challenges and best practices encountered during the response***

- ***Documenting and sharing experiences of response stakeholders***

- ***Identifying practical actions for improving existing capacities and capitalizing on best practices***

- ***Improving preparedness, readiness and response plans.***

As a means of demonstrating our commitments under IHR (2005), the MoHS will share AAR reports with MDAs, partners and neighbouring countries. Such reports would outline lessons, experiences, models and the advocacy for support for preparedness and readiness action,

Guidance for After Action Review (AAR). Geneva, Switzerland: World Health Organization

4 Building the AAR Facilitation Team

Whether undertaking a Hot Debrief, an Internal or Multi Agency AAR, the size and roles of the team will depend on the AAR format being used. In addition, if dealing with a large protracted incident the debrief might need to be conducted over a couple of days. As explained previously, the Incident Manager who has overall responsibility for the AAR and for activating the lessons identified process. In doing so, the IM will appoint a suitably trained and experienced Facilitator to undertake the required debrief. However, the IM retains responsibility for liaising with senior managers of MDAs and partners, informing them of the planned event.

The Facilitator who will act as the "**Lead Facilitator**", should be appointed to help create an open environment, promote discussions and draw out lessons learned. In cases such as a **Hot Debrief**, this could be undertaken by any Pillar Lead or the Manager for Public Health Emergencies. The Lead Facilitator is the person who leads the facilitation at the workshop and should ensure that the report is completed.

It is important to recognize that the Lead Facilitator appointed for **an AAR**, does not necessarily need to know the event in any detail and that they should be impartial. They should be external to the response and can come from within the health sector or elsewhere, including academia, humanitarian organizations or civil society. Their independence will help them unearth problems more easily if they are seen as being a neutral third party. Everyone will have a different truth to share of the same event, so individual needs, rights and views will need to be respected at all time.

Once appointed, the Facilitator will agree on the aims and objectives for the debrief with the IM and set about establishing a team and start to develop an agenda. This document contains practical aides that will support the affective delivery of the different type of debriefs to be undertaken

The size and scope of the AAR Team is very much dependent on the scale of response and the number of agencies involved. For single agency AAR, **Support Facilitators** could be coopted from Pillar or Functional Area Leads who were involved in the response. Equally, when undertaking a multiagency AAR, Support Facilitators could be co-opted from MDAs.

Support Facilitators should have a good understanding of the event under review and may have played a role in the response. The Support Facilitators are there to support the Lead Facilitator in gaining situational awareness be collating background information. This background information not only provides a common operating picture but supports the facilitation tools required for the AAR. Background information could include Outbreak Plans, Emergency Response Plans, SOPs etc. In addition, the Support Facilitators help to guide the discussions around themed areas that they are conversant with and prevent deviation beyond the planned scope and objectives.

The AAR Team should also have an **Administrator** who is able to take notes. It is important to capture comments and discussion points, ensuring that they are documented and reflected in the report. The administrator would work closely with the facilitators to ensure that all the salient points are captured.

When working in larger groups that have individual Response Pillars or Function Areas participating, it is important that they identify a note-taker who can capture group discussions for

feedback in the plenary sessions. The note-taker does not necessarily have to be a technical expert but should have some familiarity with the topic area.

When considering the makeup of the AAR Team it is important to consider individuals' capacity to influence others. It is **not advisable** to have somebody who is overbearing, or too senior as this could potentially stop participants from speaking freely or make critical remarks. Facilitators need to remain impartial and not influence group or individual feedback.

They need to be able to listen more than speak; be able to clarify and summarize key points; and be able to guide participants through the discussions or the interviews. The most important attribute for facilitators is their **interpersonal** and **communication** skills. They should be able to drive discussions and keep asking the question **why,** in supports of a root cause analysis approach.

Root cause analysis is a method used to identify the factors that led or contributed to success or failure in relation to a specific issue or problem identified. The root cause is a factor that leads directly to a particular outcome (good or bad). The removal of this factor will prevent the outcome from occurring. The purpose of conducting such analysis during an AAR is to identify and eventually address root causes, if necessary, in order to prevent negative outcomes. The purpose of the analysis is to focus on interventions that have a long-term impact rather than relying on quick fixes. Root cause analysis should be used when a problem is identified that clearly requires deep examination, or for which the cause of a challenge is not yet fully understood.

The "5 Whys method" is the simplest and most frequently used approach to root cause analysis. In essence, the facilitator repeatedly asks "why?" in order progressively to unpack causative factors, thus eventually getting to the root cause of a particular issue. This technique is most appropriate in the framework of an AAR group discussion.
Guidance for After Action Review (AAR). Geneva, Switzerland: World Health Organization

Once the AAR Team has been established, the Lead Facilitator will ensure that each team member is briefed, and their role and responsibilities conveyed. Team briefings should take place several days before the actual AAR for team members to become familiar with any guidance or background material. Team members should become familiar with this guidance document and the supportive information within.

Once the format of the AAR has been decided the Lead and Support Facilitator should work together to ensure that each Functional Area and Responding Pillar has an assigned Support Facilitator and where possible a note taker. Individual interviews may need to be conducted prior to the workshop, which should be undertaken by the Support Facilitators.

The Lead Facilitator, working with the IM and administrative support will ensure that an appropriately sized room is available and has been booked for the duration required. The Administrator would ensure that registration arrangements are in place and that any required documentation is available on the day. The participant, in all cases, need to be informed of the time and location of the debrief in a timely manner. In addition, any supportive information such as a questionnaire should be circulated well in advance of the debrief.

The Lead Facilitator will take responsibility for collating and then consolidating the outputs from all the group discussions to produce a final AAR report.

5 Preparing for an After Action Review

After Action Reviews are designed to be flexible and can be adapted to fit the event under review. Its success hinges on the ability to bring relevant response stakeholders together in an environment where they can analyse actions taken in the response in a critical and systematic fashion and identify areas for improvement.

Whilst AARs can vary in scope and format, all AARs should involve:

1) A structured review of response activities

2) An exchange of ideas and an in-depth analysis of what happened

3) Identification of what can be addressed immediately

4) Identification of what can be done in the longer term to improve responses to the next event.
Guidance for After Action Review (AAR). Geneva, Switzerland: World Health Organization

Each responding organisation should be represented by suitable individuals who took part in the response and are able to convey their organisational prospective into the AAR. Equally, each Pillar and or Functional Responding area should be represented at the debrief. It is important that Organisational / multisectoral issues are conveyed and not personal or psychological ones.

The overarching aim of the AAR is for delegates to communicate their experiences of an incident/outbreak/exercise so that lessons can be identified. In particular, the AAR is focused on identifying what worked well or not, how these practices can be maintained, improved and be shared with relevant stakeholders.

In that regard, it is vital to understand your audience in order to structure focus areas of discussion. Things are made easier if dealing with a single organisational team, who have a joint understanding of the intended systems and process to be used. Although the situation is slightly compounded, it is easily surmountable for multidisciplinary teams across Ministries, Departments and Agencies (MDAs).

Therefore, as a Lead Facilitator it might be prudent to prepare some leading/ trigger questions that can be asked across MDAs. Such questions could be themed around Organisational, Functional or Response Pillar Areas. Additional to the themed areas the AAR is focused on addressing the following six key questions:

**1. What should have happened?**	**2. What Actually happened?**	**3. What worked well?**
**4. What didn't work well?**	**5. Why was there a difference?**	**6. What are the recommendations for next time?**

In addition to these focus areas, the following categories are provided as examples of the themes/issues that will need to be covered as part of the structured debrief (this list is not exhaustive and can be added to as required):

(1) **Coordination**
a. ***Coordination within the health sector***—roles and responsibilities and coordination at administrative levels (local, regional and national)
b. ***Coordination across sectors***—with partners and, where relevant, with the international community.

(2) **Resources**
a. ***Human resources capacity***- availability of qualified and trained human resources
b. ***Relevance of plans and procedures -*** clarity in roles and responsibilities and planned actions
c. ***Financial and* material resource requirements**—availability of equipment, logistics and funds.

(3) **Communications**
a. ***Internal-*** How were the MoHS response Pillars notified
b. ***Multiagency*** – how were MDA's, WHO and Partners notified

(4) **Technical aspects**
a. Specific technical aspects related to the pillar under review.

5.1 An example of a Coordination Pillar Trigger Questions:

1) Objective observation

- What are the existing mechanisms for multisectoral coordination? How should these mechanisms be activated?
- What are the existing mechanisms for coordinating international and national partners such as the United Nations, non- and inter-governmental organizations (NGOs and IGOs), the Global Outbreak Alert and Response Network (GOARN), emergency medical teams (EMTs), etc.?

2) Analysis of gaps and contributing factors

- How did multisectoral coordination, decision-making and information and resource sharing take place during the event? Was it effective? Did it enable the health sector to have an effective role?
- How did the coordination of international and national partners (e.g. IGOs, NGOs, the UN, GOARN, EMTs, etc.) take place? Was it effective?
- Was a joint interagency or multisectoral response plan developed? If so, did this enhance the response?
- Were interagency clusters activated and operational? If so, were such clusters effective for coordinating roles and responsibilities, and for ensuring complementarity between partners?
- Were sufficient resources (human, material and financial) available for multisectoral coordination?

3) Identify areas of improvement

- What can be done to improve coordination next time?
- What can be done to improve preparedness and the response process next time?

Guidance for After Action Review (AAR). Geneva, Switzerland: World Health Organization

6 Conducting a Hot Debrief

In addition to the steps outlined above, the following pages provide practical steps to be taken for each given debrief.

A hot debrief is undertaken immediately/ or as soon as is practicable after the incident/outbreak/exercise, once a shift or the response is completed. It is important to capture the "here and now" views of team members whilst they are available, and things are fresh in their memories, before reflection.

A hot debrief can be undertaken on an individual basis or as part of a broader team discussion. For an individual team discussion, periodically throughout or at the end of a shift, the Incident Manager might ask individual Pillar Leads, their views on how things are going and to check on the wellbeing of their staff members. This type of debrief is informal and is focused on specific operations of a single team.

This type of Hot Debrief will not take a long time and should allow participants the opportunity to decompress after what could have been a difficult experience. The scope is very narrow, allowing for focused learning outcomes. In all cases, the Incident Manager would keep notes of these discussions which would feed into a structured debrief.

A more structured Hot Debrief could be held for each Response Pillar or Functional Area following a simulation exercise, emergency or an incident. In all cases, the process and methodology of debriefing are the same for extracting the required information.

In order to deliver a structured Hot Debrief, it is important to determine what technique will been used. Whether debrief on an incident/emergency or an exercise, it is crucial to remember that the debrief is a facilitated participant focused event and that execution of delivery is key. The facilitator should not hog the limelight but should be focused on hearing the views of all participants.

As previously stated, the process should not take long and should be able to elicit any immediate issues. The initial considerations for a hot debrief are:

- What is going well?
- What is not going well?
- What are the issues?
- What are the recommendations?

Each question can be posed to individuals or on a team by team basis. In all cases, it is important to give the participants some time (a small amount) to reflect on each question. However, that said, it is recognised that team discussions will take slightly longer.

In all instances, the responses will be recorded on a Hot Debrief Form/ flipchart or electronically. All the produced recommendations should be agreed at the end of the sessions and an Action Plan for implementation should be agreed.

For standardisation of approach, the following draft agenda template should be considered when undertaking a formal Hot Debrief:

6.1 – Draft Agenda Hot Debrief Template

Key Areas	Session	Key Notes
00:00hrs	**Introduction**<br><br>Purpose of the Hot Debrief<br><br>Aims and objectives	
00:10hrs	**Negative Observations**<br>List one – three negative aspects of the incident/exercise on the debrief form?	
00:20hrs	**Positive Observations**<br>List one – three positive aspects of the incident/exercise on the debrief form?	
00:30hrs	**Learning Points**<br>1) What should be done differently?<br><br>2) What would be your single most important recommendation?<br><br>3) If I was responsible for the plan my 2 priorities would be?	□
00:40hrs	**Conclusion/ Close**<br>Reminder: of internal debriefs scheduled!	

In the context of the MoHS, a Hot Debrief is intended to be a focused snappy process conducted at the end of an incident/ exercise or shift. It allows participant the opportunity to decompress and for the IM to check on their wellbeing, whilst considering any Health and Safety issues.

6.2 – Key Facilitator Notes for Hot Debrief

**Introduction**	□ aims: write them up and keep in view<br>□ explain overall approach and timings<br>□ explain housekeeping, ground rules and encourage open and honest input<br>□ all views are valid, we are not looking for group consensus or decision<br>□ say what will be done with debrief input
**Recap**	□ go for a clear visual outline - keep it simple<br>□ purpose is to stimulate thinking and provide hooks for ideas
**Ponder**	□ facilitator write up prompt questions (if possible)<br>□ use of different coloured sticky notes<br>□ keep control of time: use time evenly across all questions asked
**Sharing and discussing**	□ one person talking at a time<br>□ deal with negative views first<br>□ all should have equal opportunity to share their thoughts<br>□ use pictures / prompt diagrams to map post-it-notes<br>□ ask facilitative questions to bring out / develop points made<br>□ encourage discussion<br>□ move on to positive views for second half of the period
**summary**	□ Be concise, do not try to evaluate what has been raised
**Ponder**	□ Start of closing stage<br>□ Final prompt question
**sharing**	□ Listening to each other<br>□ Read out own words in turn, no need for lengthy explanations
**Close**	□ Thank all for their time<br>□ Reiterate what will be done with information

6.3 – Template – Hot Debrief Form

This Hot Debrief Sheet should be used following any exercise or incident. Please complete as honestly as possible.

**Name:**	
**Organisation:**	
**Contact:**	
**Name of Incident/ Exercise**	
**What was your role in the response?**	
**Summary of what did <u>not</u> so well?**	1.<br>2.<br>3.
**Summary of what <u>did</u> go well?**	1.<br>2.<br>3.
**What was your greatest challenge?**	
**What would you change if you were faced with the same situation again?**	
**What are the areas for Improvement?**	
**Have you identified any training needs from this activity (either individual or for the organisation)**	

The forms should be collated and then fed into any AAR conducted.

The following key questions should also be considered by the facilitator to support discussion flow.

6.4 – Key areas Questions

- Were the incident/exercise response objectives met?

- Does the feedback suggest that all personnel would be able to successfully compete the tasks necessary? If not, why not?

- What are the key decisions associated with each activity?

- Does feedback suggest that all personnel are adequately trained to complete the activities or tasks needed to demonstrate capability?

- Were any resource shortcomings identify that would inhibit the ability to execute activity?

- Do the current plans, policies and procedures support effective delivery of activities? Are Responders familiar with these documents?

- Do personnel from multiple agencies need to work together to perform a task, activity, or capability? If so, are the agreements or relationships in place to support the coordination required?

- What should be learned from this event? What strengths were identified for each activity?

- What areas for improvement are recommended for each activity?

The following are reminders for facilitator:

- Has the room been booked for the duration of the meeting?
- Have you got administrative support?
- Has a delegates list been drafted?
- Have the objectives been agreed with the IM and has the agenda been drafted?
- Have delegates been informed of the start time and location of the debrief?
- Are flipcharts and marker pens required?
- If needed, has somebody been identified to present the timeline of events?
- Are refreshments required, if so, have they been booked?

7 Conducting an After Action Review

The AAR is a simple but powerful tool which provides a structured process to analyse what happened, why it happened and what should be done different for the next event. AAR provide a qualitative review of actions taken in response to an incident or event. This structured format enables learning to be captured from the whole event, in a reflective way.

It is important to note that the learning from any Hot Debrief, that may have been undertaken earlier, would feed into this structured process to ensure that all lessons are captured for a single event. However, unlike a hot debrief, delegates would have had more time to reflect upon their experience and prepare for the workshop.

In cases such as a multi-agency response, it is expected that individual organisations would have had their own debrief, the outputs off which would feed into this structure debrief process. To this end, it is important that the representatives from MDA's and Partner actually took part in the response itself, not only for their situational awareness but for the added context they would bring.

The scope and format on an AAR can vary greatly and is very much dependant on the event to be reviewed. In all case, the format should be structure in a way to support the exchange of ideas whilst allowing for an in-depth analysis of what happened. The Facilitator should ensure that delegates identify actions that can be addressed immediately and those that could be improved more longer term to improve future responses.

There are three phases common to all AARs:

1. **Objective observation**: establish how actions were actually implemented, rather than how they would ideally have happened according to existing plans and procedures.

2. **Analysis of gaps and contributing factors:** identify gaps between planning and practice; analyse what worked and what did not work, and why.

3. **Identification of areas for improvement**: identify actions to strengthen or improve performance and determine how to follow-up.

In determining the scope of an AAR, it is important to recognise that the IMS used within the MoHS is based upon Functional Areas and responding Technical Pillars. As such, it is important to consider the number of Pillars and Functional Areas that are being reviewed as part of the AAR and the time that this would take. Adopting this approach, ensures that each participating response Pillar can share learning, consensus build, whilst supporting the validation of recommendations between technical working groups.

Experience has demonstrated that a work group format is most effective when reviewing multiple Pillars within the MoHS. This structured methodology is based upon group exercises, plenary discussions and interactive facilitation techniques. These sessions also lead to greater understanding of the interdependency between disciplines, response stakeholders and MDA's.

The table below provides examples of Pillars and the technical areas or functions to be considered. All of which is helpful when designing the scope of the review.

7.1 Example of Structured Pillars

PILLAR EXAMPLE	TECHNICAL AREAS/FUNCTIONS
Surveillance	• Surveillance and early warning<br>• Alerts management<br>• Surveillance information management<br>• Contact tracing
Laboratories	• Laboratory capacity for testing<br>• Specimen transportation and referral<br>• Specimen management<br>• Laboratory information management
Coordination and emergency response	• Coordination of the response at all levels (i.e. in communities, within the health sector, with other sectors and partners, and internationally)<br>• Logistics<br>• Preparedness plans<br>• Incident management system (IMS)<br>• Emergency response operations<br>• Rapid response teams (RRT)<br>• Surge capacity<br>• Resource mobilization<br>• Emergency financing mechanisms
Communication and community engagement	• Public communication<br>• Risk communication<br>• Community engagement
Case management and countermeasures	• Case management<br>• Infection prevention and control (IPC)<br>• Medical countermeasures<br>• Quarantine<br>• Immunization<br>• Safe burials<br>• Vector control and reservoir management

Guidance for After Action Review (AAR). Geneva, Switzerland: World Health Organization

As explained previously, the methodology for delivering an Internal Organisational Structured and a Multi-Agency AAR is the same. The only difference would be the scale, scope and size of the reporting areas. As such, it is important to factor this into your agenda for the day. When undertaking an internal structured AAR, it might prove advantageous to ask reporting areas to come prepared with a presentation outlining the following 6 areas:

**1. What should have happened?**	**2. What Actually happened?**	**3. What worked well?**
**4. What didn't work well?**	**5. Why was there a difference?**	**6. What are the recommendations for next time?**

7.2 Example of a half-day workshop agenda

Time	*Session*	*Facilitated by*
08:30	Arrival of participants/ registration	
08:40 – 09:20	Welcome and introduction:<br>- Opening remarks<br>- Individual Prayers<br>- Agenda and purpose	
09:20 – 09:50	Review of incident/exercise:<br>- Pre-event preparation<br>- Summary of event and outputs<br>- Summary of event feedback	
09:50 – 10:30	Participant group discussions:<br>- Split into Functional/Pillar/ Organisational Area<br>- Consider/ develop presentations based on AAR 6 key questions	Support Facilitators
10:30 – 10:50	***TEA & COFFEE BREAK***	***All***
10:50 – 11:30	Focus group discussions continuation:<br>- Group work	Support Facilitators
11:30 – 12:30	Group feedback and facilitated discussion	
12:30 – 12:45	Event Action Plan/next steps	Lead Facilitator
12:45	Final remarks and closing:<br>- IM<br>- Lead Facilitator	
13:00	***LUNCH***	***All***

The agenda above provides a draft example and should tailored to suit individual needs. Time should be extended or shortened depending on the number of Response Pillars and Functional Areas under review. A half day workshop should provide enough time to review between 4-5 functional areas effectively.

7.3 Key Notes for AAR Debrief Facilitators

**Introduction**	aims: write them up and keep in view<br>explain overall approach and timings<br>explain ground rules and encourage open and honest input<br>all views are valid, we are not looking for group consensus or decision<br>say what will be done with debrief input
**Recap**	go for a clear visual outline - keep it simple<br>purpose is to stimulate thinking and provide hooks for ideas
**Ponder**	facilitator write up prompt questions (if possible)<br>use of different coloured sticky notes<br>keep control of time: use time evenly across all questions asked
**Sharing and discussing**	one person talking at a time<br>deal with negative views first<br>all should have equal opportunity to share their thoughts<br>use pictures / prompt diagrams to map post-it-notes<br>ask facilitative questions to bring out / develop points made<br>encourage discussion<br>move on to positive views for second half of the period
**summary**	Be concise, do not try to evaluate what has been raised
**Ponder**	Start of closing stage<br>Final prompt question
**sharing**	Listening to each other<br>Read out own words in turn, no need for lengthy explanations
**Close**	Thank all for their time<br>Reiterate what will be done with information

7.4 Template – AAR Debrief Form

This AAR Debrief Sheet should be used following any exercise or incident. Please complete as honestly as possible.

<table>
<tr><td colspan="2">Name: Organisation: Contact:</td></tr>
<tr><td colspan="2">Date Location of Incident</td></tr>
<tr><td colspan="2"></td></tr>
<tr><td>Name of Incident/ Exercise</td><td></td></tr>
<tr><td>Your Role in the response?</td><td></td></tr>
<tr><td>What were you expected to do?</td><td></td></tr>
<tr><td>What did you do?</td><td></td></tr>
<tr><td>Did you refer to any plans or procedures? If so which ones? If not why not?</td><td></td></tr>
<tr><td>What was your greatest concern?</td><td></td></tr>
<tr><td>Summary of what went well</td><td>1.
2.
3.</td></tr>
<tr><td>Summary of what did not go well</td><td>1.
2.
3.</td></tr>
<tr><td>What would you change if you were faced with the same situation again</td><td></td></tr>
<tr><td rowspan="4">What areas for improvement are there in terms of?</td><td>Your role:</td></tr>
<tr><td>The incident/exercise generally:</td></tr>
<tr><td>Your organisational response:</td></tr>
<tr><td>The multi-agency response:</td></tr>
<tr><td>Have you identified any training needs from this activity (either individual or for the organisation)</td><td></td></tr>
<tr><td>Other</td><td></td></tr>
</table>

7.5 Example Trigger Questions

The following sections provide as examples of trigger questions that can be considered by participating pillars. The questions are framed to help facilitators keep discussions flowing.

Each scenario will differ dependent upon the event being reviewed, so the list should be considered as none exhaustive. Flooding, chemical events, natural disasters, Zoonotic diseases etc, will all have different sets of trigger questions accordingly.

PILLAR EXAMPLE	EXAMPLE QUESTIONS
**Case management**	• How were cases and fatalities managed during the emergency?<br>• How were patients transported/referred between healthcare facilities?<br>• How was the coordination of case/fatality management undertaken between sectors and partners?<br>• Were the necessary equipment/material/resources available for case management and personal protection?<br>• What was the role of the public sector and/or other actors in case management?<br>• How was the case management financed? Was it free for patients?
**Infection prevention and control**	What IPC measures were implemented to protect health care workers, patients (in-patients and out-patients) and communities? Were they sufficient?<br>• Were the IPC measures that were implemented during the emergency effective in preventing infection in a health care setting or in the community?<br>• How was waste managed in health structures and in the community following funerals?<br>• Were enough resources available to protect staff from infection (e.g. personal protective equipment/PPE), for waste disposal, and for decontamination?<br>• How was coordination with other sectors—including the private sector— ensured in the implementation of IPC measures in health care facilities and communities, and for water and sanitation for health (WASH) activities?
**Logistics**	•How were supply chains managed during this emergency?<br>• Was the prepositioning of essential material effective in enabling a timely and efficient response?<br>• Were enough resources (human, material and financial) available to provide logistics support during the event?<br>• How was fleet management undertaken during the response?<br>• Were there other partners or sectors involved in delivering logistics services? What were their role(s) and how was it coordinated and managed?<br>• How did the emergency procurement system function?

7.5.1 Example Trigger Questions

PILLAR EXAMPLE	EXAMPLE QUESTIONS
**Surveillance**	• How did surveillance and/or alert systems detect the event?<br>• How much time was taken between the onset and the detection of the event?<br>• What helped in early detection or what prevented early detection?<br>• Were there sufficient resources (human, material and financial) to undertake surveillance and early warning activities?<br>• How was epidemiological data analysed and used to enable a response?<br>• How did partners or other sectors contribute to surveillance and early warning? How was information shared?<br>• How were surveillance activities adapted or reinforced through the course of the response?<br>• How did the surveillance system detect the end of the outbreak/end of the emergency situation?<br>• Did the event identify any weaknesses or gaps in the collection, storage, transmission, or analysis of surveillance data?<br>• How did surveillance for the event/pathogen change during the response (e.g. from aggregate reporting to case-based surveillance)?<br>• What were barriers to effective contact tracing (where applicable)?<br>• How was the risk of the event assessed? By who and when?<br>• How was the result of the risk assessment used? Did it have an impact on the management of the response?<br>• How did the assessment findings help to plan for the response effort?
**Laboratory**	• What is the laboratory turnaround time (i.e. how quickly the samples are collected, tested and reported back)?<br>• What was the process for laboratory confirmation?<br>• How was information coming from the laboratories managed?<br>• Were plans and SOPs for laboratory testing adequate to respond to the event?<br>• Were there sufficient resources (human, material and financial) available to provide consistent laboratory support during the outbreak?<br>• Were there any issues involved in the collection, management, and transportation of specimens?<br>• How did coordination and information sharing with other laboratories in the health sector and in other sectors function?<br>• How was the international reference laboratory involved in confirming the event?<br>• Did any accident or other biosafety incident occur? If yes, what was the cause?

7.5.2 Example Trigger Questions

PILLAR EXAMPLE	EXAMPLE QUESTIONS
**Coordination**	• How was the coordination of response actions at different administrative levels (local, regional and national) undertaken during the event?<br>• Were sufficient resources (human, material and financial) available for multisectoral coordination at all levels?<br>• Were existing contingency/response plans for this emergency effective in identifying actions, making decisions and communicating information?<br>• How was finance management undertaken during the event?<br>• How was coordination with donors managed during the event?<br>• How was information managed during the emergency? What information products were developed?<br>• How did the coordination of international and national partners (UN, NGOs, GOs, IGOs etc.) take place?<br>• Was a joint inter-agency/multisectoral response plan developed? How did this contribute to enhancing the response?<br>• Was the health cluster activated and operational?<br>•Was it effective for coordinating roles and responsibilities and for ensuring complementarity between partners? (This is particularly relevant in humanitarian emergency settings).<br>• How did the preparedness and response plan help?<br>• Identify the areas in which preparation for this event was most successful.
**Vector surveillance and control**	• What vector control measures were implemented during the emergency, and how did this impact the evolution of the outbreak?<br>• How effectively was the integrated vector management plan implemented?<br>• How were communities communicated with during vector control activities?<br>•Did communities accept and support the vector control strategy?<br>• How was intersectoral coordination/collaboration managed? How did this contribute to the efficiency of vector control measures?<br>• Were sufficient resources available and accessible for vector control activities?<br>• Were resistance patterns to the chemical products used for vector control detected?<br>•How was resistance monitored and managed?

7.5.3 Example Trigger Questions

PILLAR EXAMPLE	EXAMPLE QUESTIONS
**Communication and community engagement**	• How were risk communication activities and messages coordinated between levels of the health system (local, regional and national)?<br>• How was public communication conducted during the emergency?<br>•Was a specific communication plan developed?<br>• Did the population that was in greatest need of receiving communication messages effectively receive those messages? If not, why? How do we know?<br>• Were enough resources available to conduct risk communication community mobilization?<br>• How were communication activities and messages coordinated with other sectors and partners?<br>• How was risk communication monitored during the emergency?<br>• How were rumours and misinformation identified, and what measures were taken to counter them?<br>• How effective was public communication for building trust with the public and managing emerging public concerns?

Guidance for After Action Review (AAR). Geneva, Switzerland: World Health Organization

8 Example of an AAR PowerPoint Slide Show

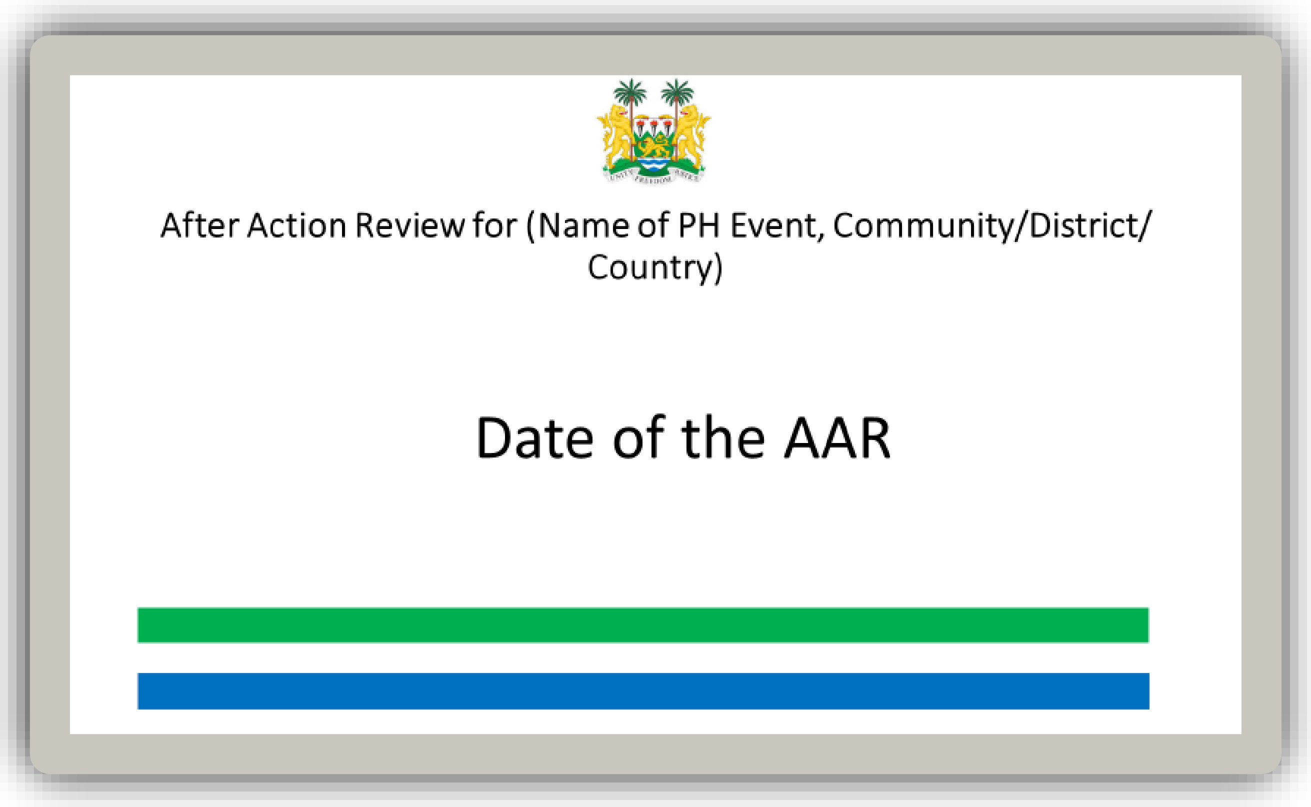

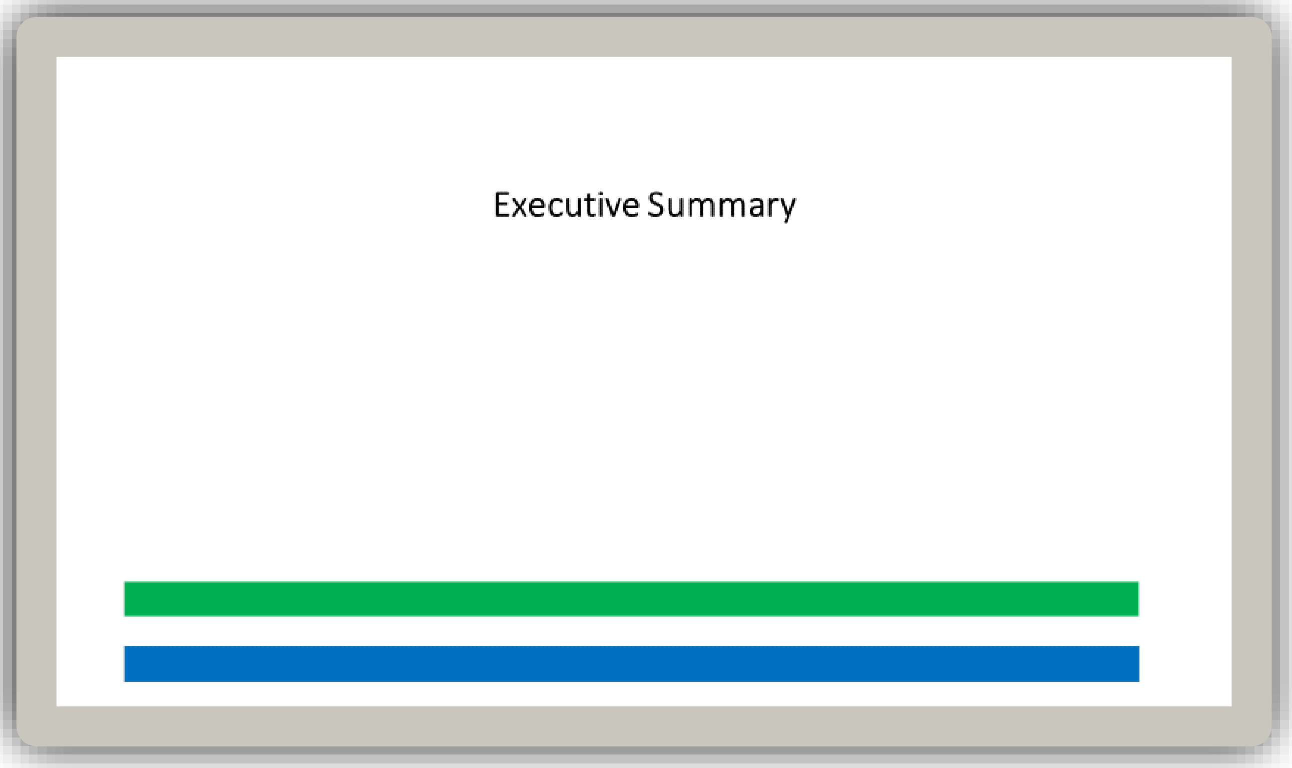
Executive Summary

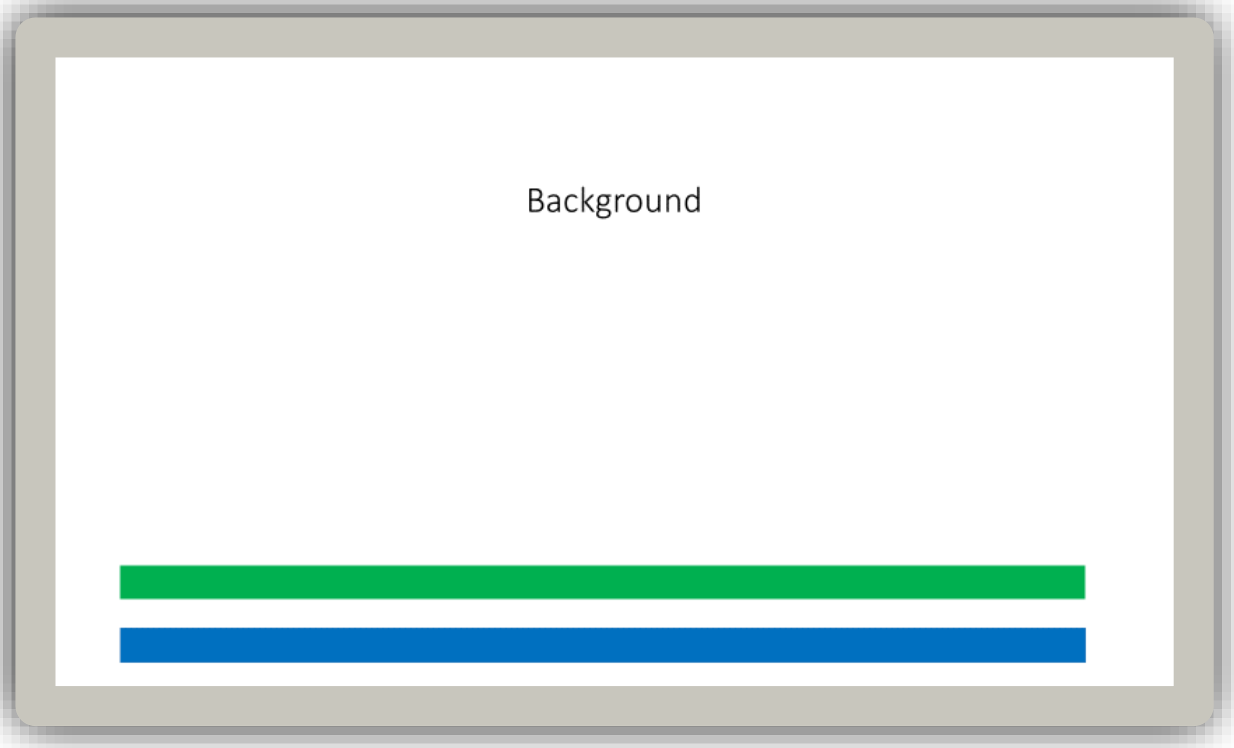
Background

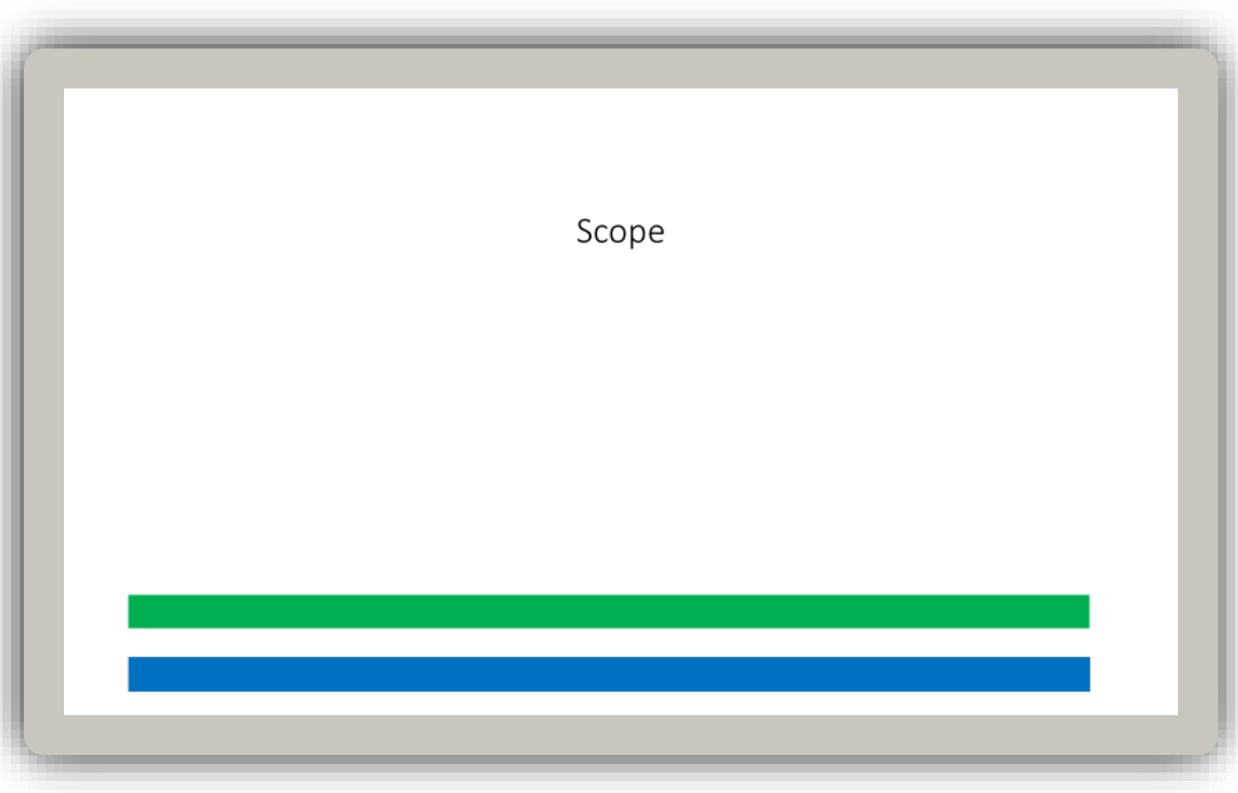
Scope

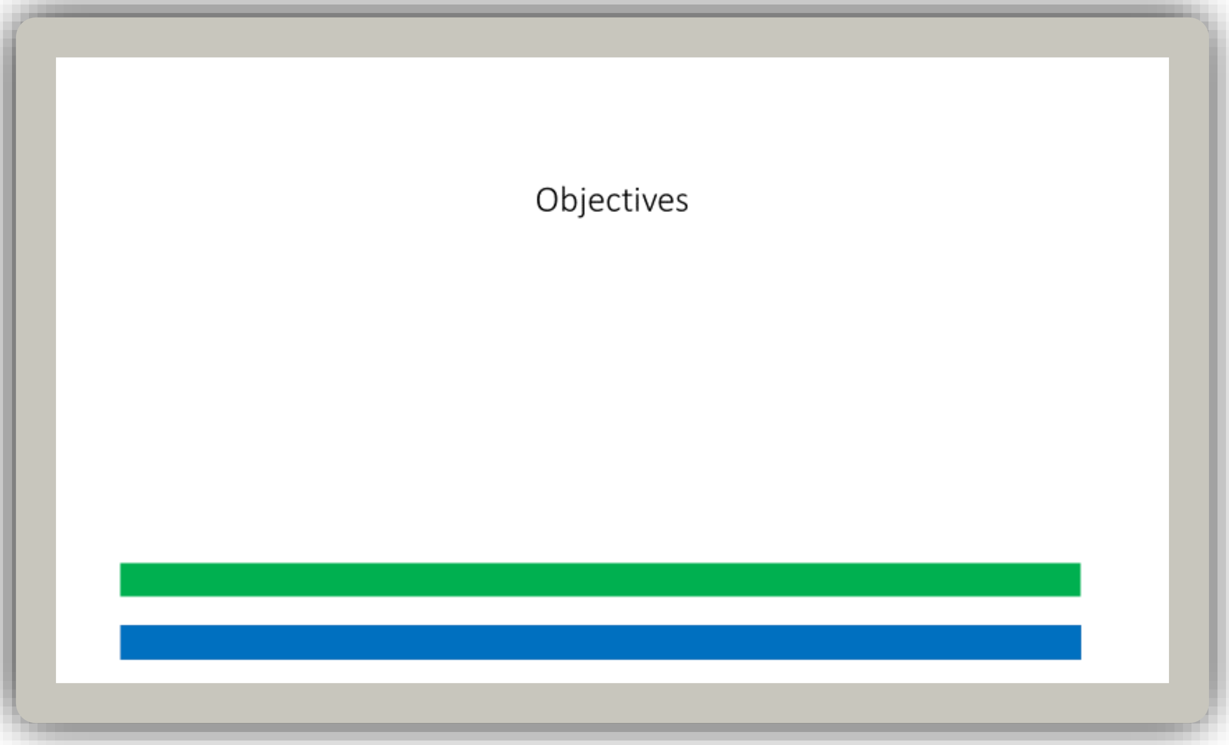
Objectives

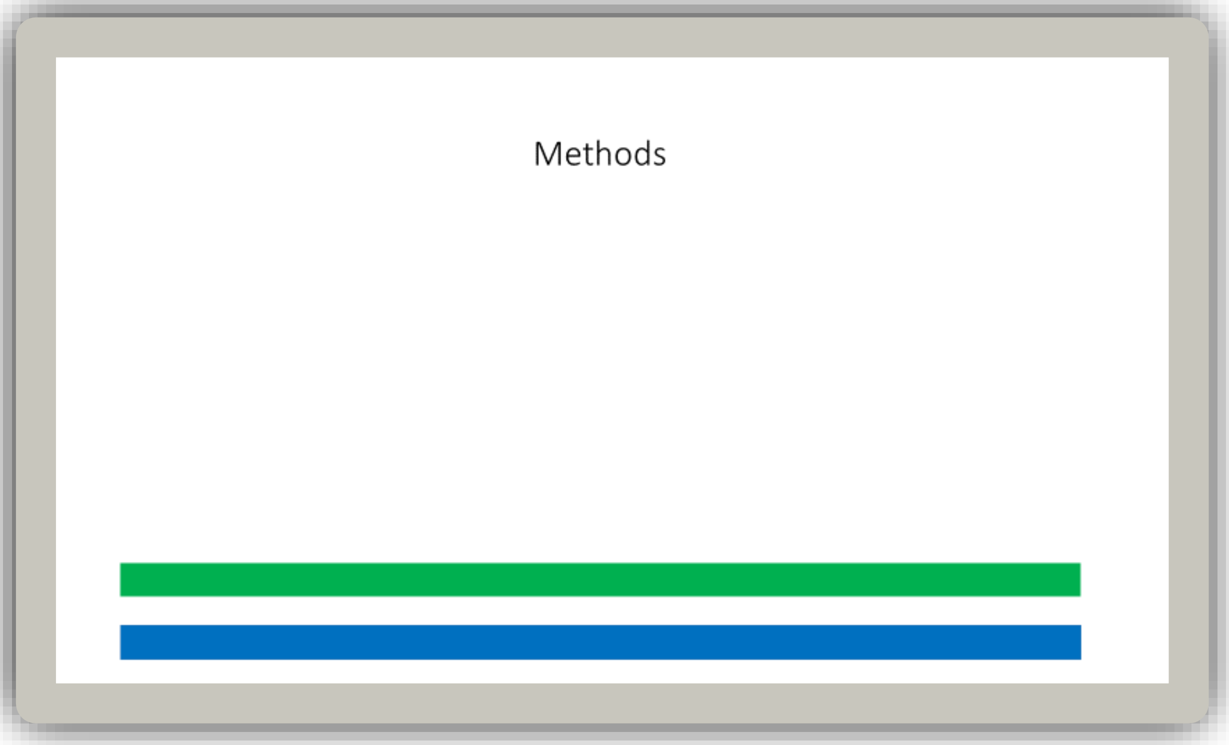
Methods

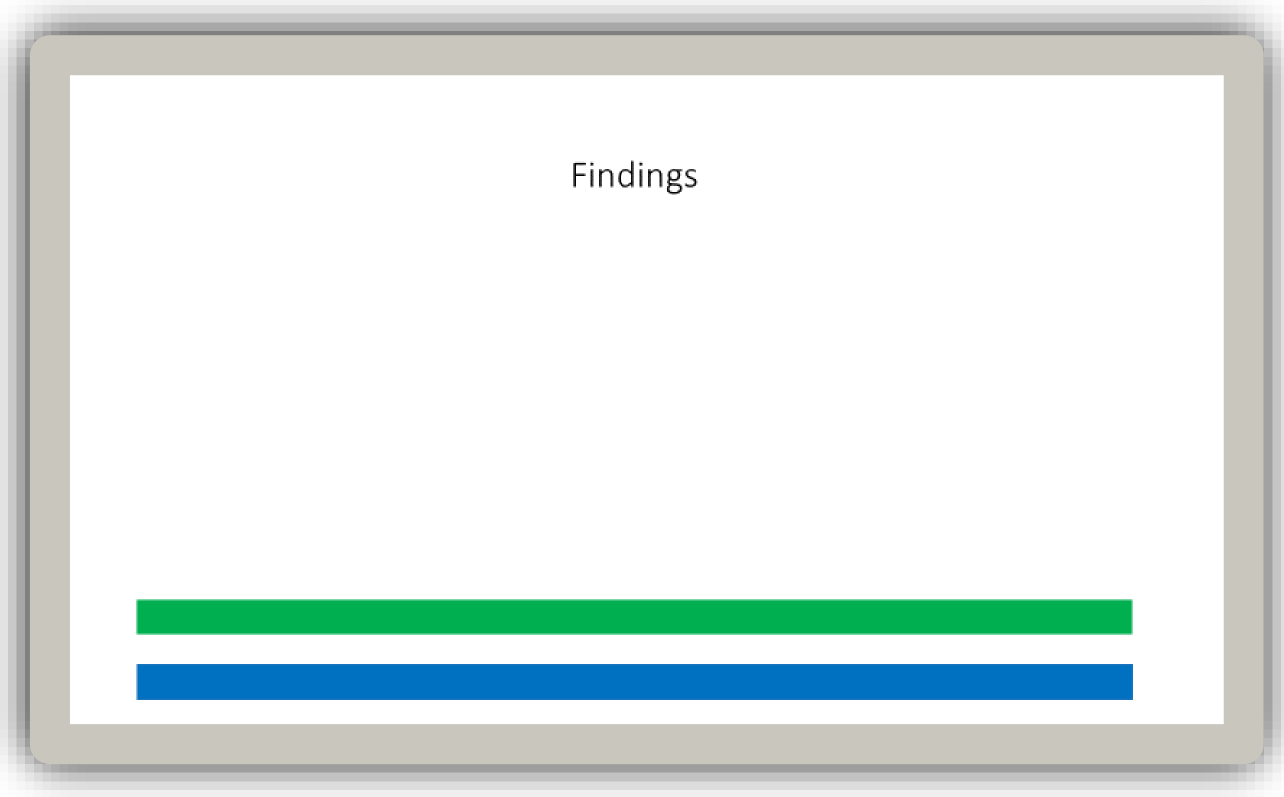
Findings

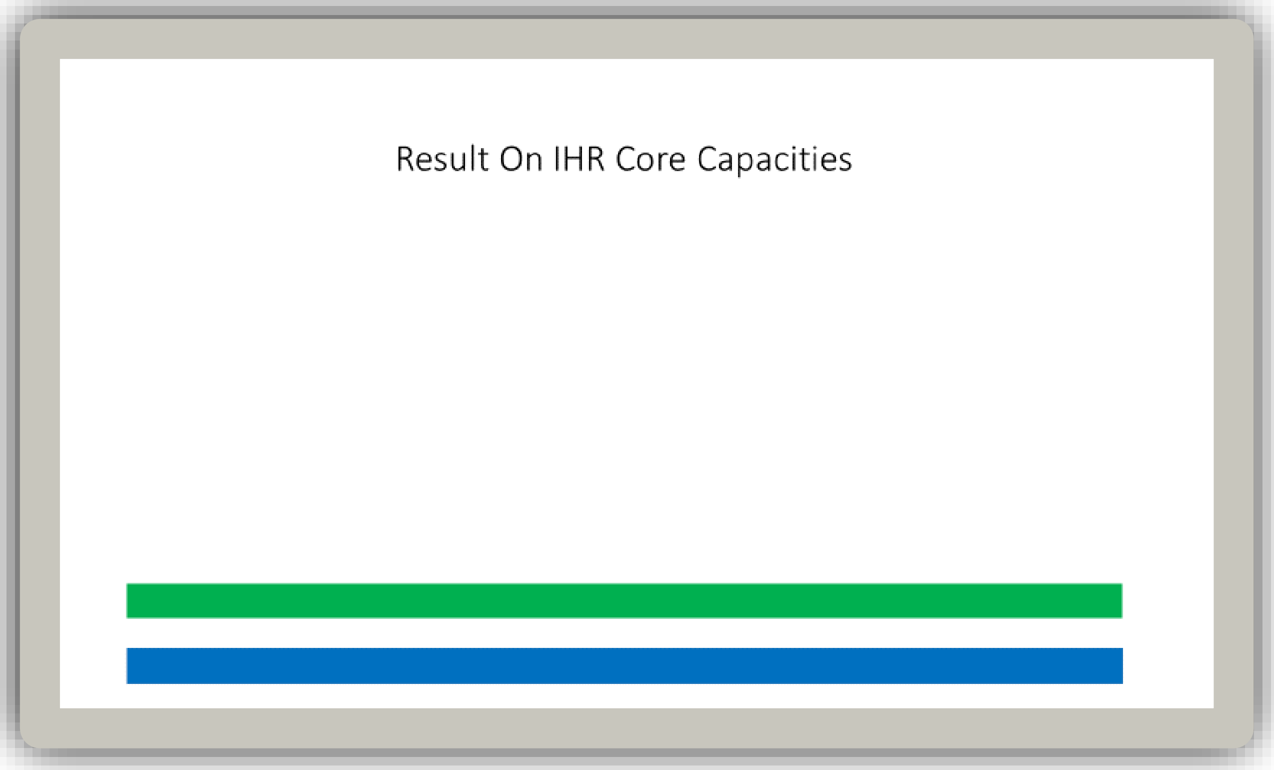
Result On IHR Core Capacities

Recommendations

Way forward

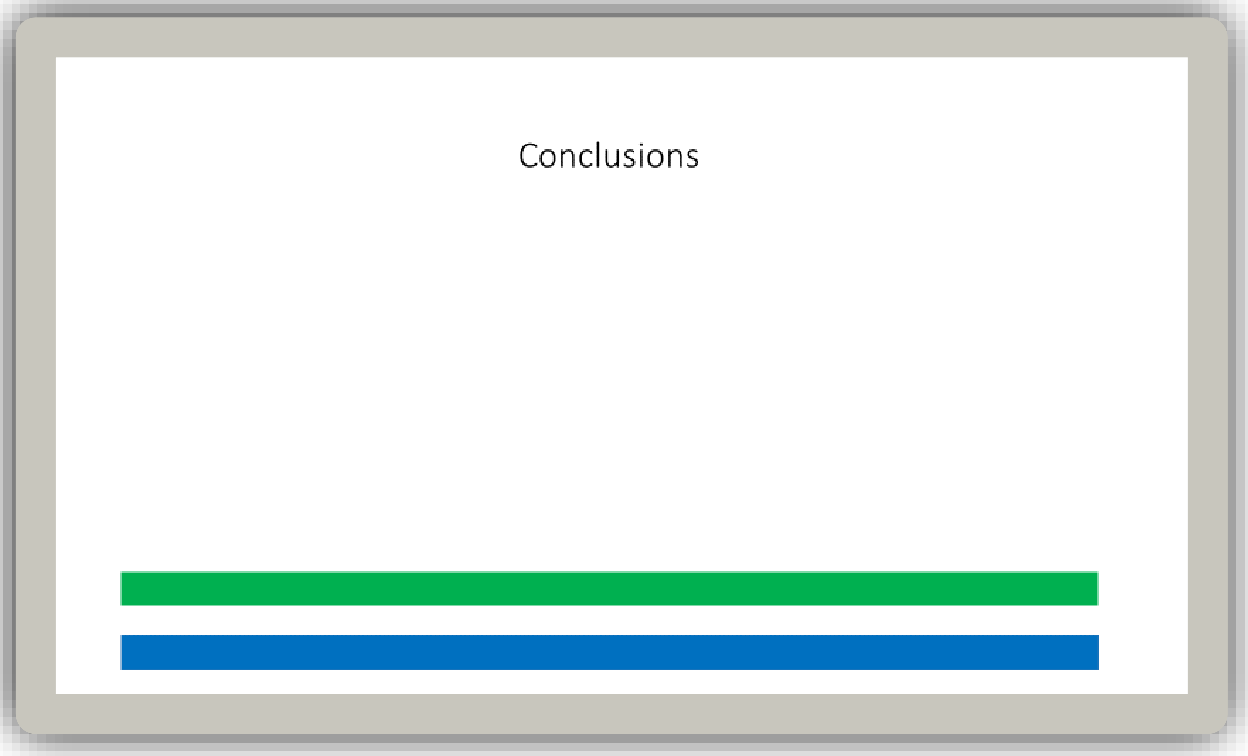
Conclusions

9. Evaluation of IHR (2005) core capacities

Whilst conducting an AAR an opportunity is afforded, whereby participants could review the extent in which selected IHR core capacities were used during the response. A goal-based evaluation with specific qualitative ratings tool is used. These ratings are as follows:

- P = performed without challenges
- S = performed with some challenges
- M = performed with major challenges
- U = unable to be performed.

To guide participants, definitions of the different ratings are provided in Annex A.
Table 9.1 presents IHR (2005) capacities and examples of evaluation criteria that can be used to identify the extent to which given capacities performed during the response.

9.1 IHR Capacity and Indicators

IHR capacities and indicators	Examples of evaluation tasks/objectives	Evaluation ratings			
		P	S	M	U
**C1: Legislation and financing**					
Legislation, laws, regulations, policies, administrative requirements or other government instruments to implement the IHR	Appropriate legislation, laws, and policies were in place and could be effectively used				
Financing for the implementation of IHR capacities	A budget was available for the implementation of IHR capacities				
Financing mechanism and funds for timely response to public health emergencies	A financing mechanism was in place that allowed for the timely flow of funds at all necessary levels				
**C2: IHR coordination and national IHR focal point functions**					
National IHR focal point functions under IHR	The National IHR Focal Point was accessible when needed and could carry out IHR functions effectively				
Multisectoral IHR coordination mechanisms	A multisectoral IHR coordination mechanism was in place and effective				
**C3: Zoonotic events and the human-animal interface**					
Collaborative effort on activities to address zoonoses	Animal and public health sectors were able to work effectively together at all necessary levels				
**C4: Food safety**					
Multisectoral collaboration mechanism for food safety events	A coordination mechanism was in place between the International Food Safety Authorities Network (INFOSAN) focal point and the national IHR focal point, and was effective for multi-sectoral coordination				
**C5: Laboratory**					
Specimen referral and transport system	Specimens collected from any level (health facilities, hospitals, etc.) reached the appropriate testing laboratory in a timely fashion				
Implementation of a laboratory biosafety and biosecurity regime	The capacity was in place to identify, hold, secure and monitor dangerous pathogens in appropriate facilities				
Access to laboratory testing capacity for priority diseases	Specimens from all levels were tested appropriately and results were available in a timely fashion				

IHR capacities and indicators	Examples of evaluation tasks/objectives	Evaluation ratings			
		P	S	M	U
**C6: Surveillance**					
Early warning function: indicator- and event-based surveillance	Surveillance data was collected at all levels and compiled, analyzed, and interpreted to guide the response				
Mechanism for event management (verification, risk assessment, analysis investigation)	An effective system was in place to verify, assess, and investigate events				
**C7: Human resources**					
Human resources for the implementation of IHR core capacities	An effective workforce was in place to prepare for, prevent, detect and respond to all hazards at all necessary levels				
**C8: National health emergency framework**					
Planning for emergency preparedness and response mechanism	The multi-hazard preparedness plan was tested and effective during the response or exercise				
Management of health emergency response operations	The emergency operations centre was activated quickly, using effective protocols				
Emergency resource mobilization	Necessary supplies, including personal protective equipment, medications, vaccines, etc., could be mobilized to the necessary levels in a timely way				
**C9: Health service provision**					
Case management capacity for all hazards	Sufficient numbers of trained healthcare workers and adequate medical supplies were in place to manage patients safely				
Capacity for infection prevention and control and radiation decontamination	Healthcare workers were trained in infection prevention and control and radiation decontamination at the necessary levels and had the necessary protective equipment				
Access to essential health services	Suspected case patients at all levels could access and utilize the required outpatient and inpatient services				
**C10: Risk communication**					
Capacity for emergency risk communication	Information to address community concerns, rumours and appropriate public health practices was effectively communicated to the public, and a feedback mechanism was in place to understand and address rumours, perceptions, and misconceptions				

IHR capacities and indicators	Examples of evaluation tasks/objectives	Evaluation ratings			
		P	S	M	U
**C11: Points of entry**					
Core capacity requirements at all times for designated airports, ports and ground crossings	Points of entry were appropriately designated and had the capacity to provide medical services and diagnostics with adequate staff and resources				
Effective public health response at points of entry	Existing contingency plans for public health emergencies at points of entry were effectively used to respond to the event				
**C12: Chemical events**					
Resources for detection and alert	The poison information service effectively detected the event, and laboratory capacity to confirm the chemical event was in place				
**C13: Radiation emergencies**					
Capacity and resources	Surveillance to detect potential radiation emergencies was in place, as were the needed coordination mechanisms and resources (including human resources) to respond				

Guidance for After Action Review (AAR). Geneva, Switzerland: World Health Organization

10 AAR Report

The AAR and the Final Report is an important part of the MoHS assurance process in compliance of the IHR 2005. The Final Report provides the evidence for further capacity building to strengthen country preparedness for public health related risks and threats.

The Report and the recommendations form a critical part in translating Lessons Identified, through action planning, into lessons learnt through corrective development. Once the Final Report has been drafted it would be shared with the IM, agreed and the circulated to relevant MDA's and Partners.

The linkages between the IHR Monitoring and Evaluation Framework and the AAR are outlined in the box below.

10.1 Links to the IHR MEF

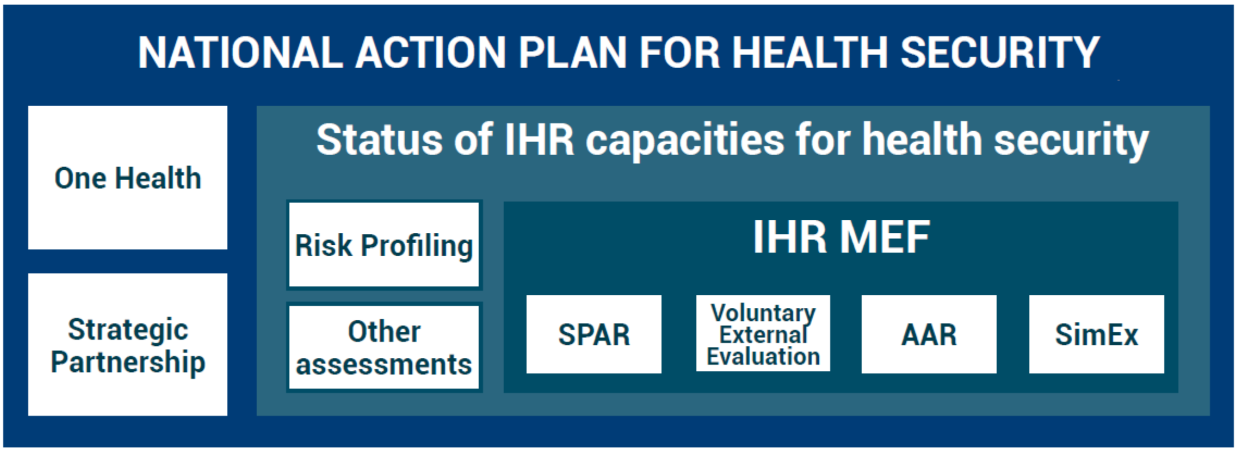

Implementation of the International Health Regulations (2005):

The Lead Facilitator will ensure that all the notes debrief notes are collated and integrated into the final report. The Lead Facilitator will work with the team of Support Facilitators to ensure the content and context within the Report is reflective of the agreed outcomes and recommendations. For ease of process and standardisation, the AAR Final Report should be comprised of the following headings:

1. Executive summary
2. Background on emergency under review
3. Scope and objective of review
4. Methods
5. Findings
6. Key activities
7. Next steps
8. Conclusions.

WHO Guidance for After Action Review (AAR)

Section 10.2 below contains an AAR Report Template

10.2 AAR Report Template

Title:

After Action Review for [NAME OF THE PUBLIC HEALTH EVENT] [COUNTRY]

Date of After Action Review: [DD/MM/YYYY]

This template should be used by the designated report writer to document and structure discussions during the After Action Review and highlight the analysis and recommendations arising from the review. This report should be shared with team members for their comments before broader circulation for knowledge sharing purposes.

---

1. EXECUTIVE SUMMARY

Briefly summarize the key points of the report in this section, which can be shared as a stand-alone document for interested stakeholders and senior management. Include:
• Brief description of the event
• Summary of discussions, including notable best practices and challenges identified
• Conclusions and recommendations.

2. BACKGROUND ON EMERGENCY

Summarize the key characteristics of the event as well as any contextual details that are relevant to provide an overview of what happened. Include:
• Timeline of the event (date of onset, key milestones, etc.)
• Number of cases, hospitalizations, deaths
• Relevant graphs/illustrations (e.g. epi curve) if necessary
• How the event was detected via existing systems
• Summary of the response
• Geographic/political/socio-economic/environmental factors that played an impact.

3. SCOPE AND OBJECTIVE OF REVIEW

Describe the rationale for organizing a review of this event.
Identify the scope and objectives of the AAR.
Identify the target focus areas of the review, and mention whether this is a sub-report of a larger review or a stand-alone report.

4. METHODS

Describe the method and approach behind the review, including:
• The format of the review (debrief, working groups, key informant interviews, mixed method)
• Participating organizations/municipalities/districts
• Description of reference materials used (can be attached as an annex).

5. FINDINGS

This is the key part of the report. Describe the discussions covered in the review, structured according to the response pillars reviewed. Focus on what actually happened, and the deeper systems/issues that explain why it happened (i.e. the root causes). Recommendations should be made both for institutionalizing and/or maintaining best practices, and for addressing challenges.

5.1 Timeline of outbreak (if applicable)

If the AAR included the development of a timeline, this section should present the timeline and highlight the key milestone dates of the response under review.

5.2 Pillar 1

Description of the response pillar and the major milestones and issues encountered. This pillar can combine several specific technical areas and/or functions.

This description should include a short narrative on the key issues within each function of the pillar in order to frame the findings in tables that lay out the following:

• Observations – best practices, impacts and enabling factors

• Observations – challenges, impacts and limiting factors

The new capacities developed under this pillar during the response should be highlighted.

5.3 Pillar 2

Description of the response pillar and the major milestones and issues encountered. This pillar can combine several specific technical areas and/or functions.

This description should include a short narrative on the key issues within each function of the pillar in order to frame the findings in tables that lay out the following:

• Observations – best practices, impacts and enabling factors

• Observations – challenges, impacts and limiting factors

The new capacities developed under this pillar during the response should be highlighted.

5.4 Pillar 3

Description of the response pillar and the major milestones and issues encountered. This pillar can combine several specific technical areas and/or functions.

This description should include a short narrative on the key issues within each function of the pillar in order to frame the findings in tables that lay out the following:

• Observations – best practices, impacts and enabling factors

• Observations – challenges, impacts and limiting factors

The new capacities developed under this pillar during the response should be highlighted.

5.5 Pillar 4

Description of the response pillar and the major milestones and issues encountered. This pillar can combine several specific technical areas and/or functions.

This description should include a short narrative on the key issues within each function of the pillar in order to frame the findings in tables that lay out the following:

• Observations – best practices, impacts and enabling factors

• Observations – challenges, impacts and limiting factors

The new capacities developed under this pillar during the response should be highlighted.

5.6 Pillar 5

Description of the response pillar and the major milestones and issues encountered. This pillar can combine several specific technical areas and/or functions.

This description should include a short narrative on the key issues within each function of the pillar in order to frame the findings in tables that lay out the following:

• Observations – best practices, impacts and enabling factors

• Observations – challenges, impacts and limiting factors

The new capacities developed under this pillar during the response should be highlighted.

5.7 Pillar 6

Description of the response pillar and the major milestones and issues encountered. This pillar can combine several specific technical areas and/or functions.

This description should include a short narrative on the key issues within each function of the pillar in order to frame the findings in tables that lay out the following:

• Observations – best practices, impacts and enabling factors

• Observations – challenges, impacts and limiting factors

The new capacities developed under this pillar during the response should be highlighted.

6. RESULTS OF THE EVALUATION OF IHR (2005) CORE CAPACITIES PERFORMANCE DURING THE RESPONSE

Immediately after identifying the best practices and challenges of the response under review, the summary of results of the goal-based evaluation of IHR (2005) core capacities performance should be presented in this chapter.

7. KEY ACTIVITIES

Include all key activities/ recommendations identified during the AAR.

8. NEXT STEPS

Include a summary of the participants' discussions related to the strategy for implementing the activities identified during the AAR.

9. CONCLUSIONS

Summarize the discussions, key points, and analyses discussed above. Include how recommendations will be implemented and tracked and specify the accountability for implementation.
Include results of the AAR evaluation and propose any improvement to methodologies for conducting the AAR.

10. ANNEXES

Annex 1: Post-AAR action plan (see AAR toolkit for template)
Annex 2: List of participants and AAR team WHO Guidance for After Action Review (AAR)

Guidance for After Action Review (AAR). Geneva, Switzerland: World Health Organization

11 AAR Action Plan

The development on an Action plan is to ensure that each recommendation highlighted during the AAR, has an assigned owner who becomes responsible for its implementation. The action plan builds upon areas which worked and seeks to address all identified gaps. A timeline for implementation is included in the action plan, to ensure that actions are completed in a timely manner.

The Action Plan will have prioritised activities reflecting their urgency for implementation. The actions will be prioritised in such a way to reflect their implementation over the short, medium or long term. There may be some activities that require a longer-term implementation period which could be included into other planned activities. An example of this is the National Action Plan for Health Security (NAPHS), which is a comprehensive, multisectoral and collaborative plan to increase preparedness for public health threats.

The implementation of each recommendation should be closely monitored, which is a role that could be undertaken during the weekly Emergency Preparedness Resilience and Response Group (EPRRG) meetings.

11.1 Action Plan Template

The template below and the headings under the 'Lessons identified' column are suggestions which may need to be changed to suit your needs.

**Lesson Identified**	**Level of Operation** Is the lesson aimed at Strategic, Tactical or Operational?	**Recommendation**	**Suggested Owner**	**Target Date**	**Priority** Red/ Amber/ Green
**1. Case Management**					
a)					
**2. Infection prevention and control (IPC)**					
a)					
**3. Medical countermeasures**					
a)					
**4. Quarantine**					
a)					
**5. Immunization**					
**a)**					
**6. Safe burials**					
a)					
**7. Vector control and reservoir management**					
a)					

12 Annex A DEFINITION OF AAR INDICATORS GOAL-BASED EVALUATION RATINGS

EVALUATION RATING	DEFINITION
Performed without challenges **(P)**	The targets and critical tasks associated with the core capability were completed in a manner that achieved the objective(s) and which did not negatively impact the performance of other activities. Performance of this activity did not contribute to additional health and/or safety risks for the public or for emergency workers, and it was conducted in accordance with applicable plans, policies, procedures, regulations, and laws.
Performed with some challenges **(S)**	The targets and critical tasks associated with the core capability were completed in a manner that achieved the objective(s) and did not negatively impact the performance of other activities. Performance of this activity did not contribute to additional health and/or safety risks for the public or for emergency workers, and it was conducted in accordance with applicable plans, policies, procedures, regulations, and laws. However, opportunities to enhance effectiveness and/or efficiency were identified.
Performed with major challenges **(M)**	The targets and critical tasks associated with the core capability were completed in a manner that achieved the objective(s), but some or all of the following were observed: demonstrated performance had a negative impact on the performance of other activities; performance contributed to additional health and/or safety risks for the public or for emergency workers; and/or performance was not conducted in accordance with applicable plans, policies, procedures, regulations, and laws.
Unable to be performed **(U)**	The targets and critical tasks associated with the core capability were not performed in a manner that achieved the objective(s).

Guidance for After Action Review (AAR). Geneva, Switzerland: World Health Organization

13 Sources

Collison, C. and G. Parcell (2001) Learning to Fly, Oxford: Capstone.

Guidance for After Action Review (AAR). Geneva, Switzerland: World Health Organization; 2018. Licence: CC BY-NC-SA 3.0 IGO.

Implementation of the International Health Regulations (2005): Report of the Review Committee on Second Extensions for Establishing National Public Health Capacities and on IHR Implementation. Geneva: World Health Assembly; 2015 http://apps.who.int/gb/ebwha/pdf_files/WHA68/

NHS Website: https://kfh.libraryservices.nhs.uk/knowledge-management/

Whiffen, P. (2001) 'Seizing Learning Opportunities at Tearfund', Knowledge Management Review, November/December.

20180119 PHE Debriefing and Lessons Identified process_Guidance Final v03.00 ERRRDG180.pdf
