# Supplementary material for: After action review of the response to an outbreak of Lassa fever in Sierra Leone, 2019: Best practices and lessons learnt
Source: PLoS Negl Trop Dis. 2022 Oct 5;16(10):e0010755. doi: 10.1371/journal.pntd.0010755 (PMC9534430; doi:10.1371/journal.pntd.0010755)
Supplement: S2 File — (PDF) [file pntd.0010755.s002.pdf]

AFTER ACTION REVIEW FOR THE LASSA FEVER RESPONSE IN TONKOLILI DISTRICT, NOVEMBER 2019

Pillar:
Presenter:
Date:

Role in the Response:

What were you expected to do?

What did you do?

Did you refer to any Plans or Procedures? If so which ones? If not why not ?

What were your greatest concerns

what went well:

what did not go well:

What would you like to change if you were faced with the same situation again

What areas for improvement are there in terms of your ROLE

What areas for improvement are there in terms of the Incidence/Exercise generally:

What areas for improvement are there in terms of your Organizational response:

What areas for improvement are there in terms of the multi-agency response

Have you identified any training needs from this activity (either individual or the organization)
