# Supplementary material for: After action review of the response to an outbreak of Lassa fever in Sierra Leone, 2019: Best practices and lessons learnt
Source: PLoS Negl Trop Dis. 2022 Oct 5;16(10):e0010755. doi: 10.1371/journal.pntd.0010755 (PMC9534430; doi:10.1371/journal.pntd.0010755)
Supplement: Attachment — Submitted filename: Comments_AAR.docx [file pntd.0010755.s003.docx]

The manuscript is based on an after-action review (AAR) of a Lassa fever outbreak in Sierra Leone as a mechanism for improving on preparedness and response actions to public health outbreaks. The AAR is an important evaluation of disease outbreak response and documenting such findings could be useful in countries with similar contexts.

Major comment

My main concern is that the manuscript does not follow appropriately or provide the information expected in each section of a research article. It seems to be a written as a report which was then loosely attempted to be turned into a manuscript.

Other comments

Abstract: The authors state that the outbreak was caused by a breach of IPC protocols. No evidence is provided in the results to back this assertion.

Line 66: It is not apparent to the reader that the simulation exercise and capacity assessments the authors refer to are among the four components of the IHR monitoring framework

Line 68: Are AARs part of organizational learning? That needs to be clarified so that the paragraph can have coherence

Line 76: Use the appropriate citation style for the journal

Line 92: Give a reference for the sentence

Line 90 to 104: The two paragraphs are redundant and repetitive. The rationale for AARs is already stated in the introductory paragraph.

Line 116: Incident not incidence

Line 121: Wasn’t the outbreak occurrence already known after the notification from Netherlands?

Line 129 to 143: This description of the response should be in the results section.

Line 171: This subsection should be made brief and moved to before the incident description

Line 183: The table should be moved to methods

Line 190-193: Did the authors use the WHO guidance on AAR as well?

Line 196: What were the considerations or qualities of the lead facilitator? Experience in AAR?

Line 204: What did the presentation template for the pillar discussions contain? Alternatively include it as a supplementary document.

Line 206: Give a reference for the document. Were the participants trained on conducting AAR besides being provided with the document.

Line 229: The coordination description is already stated in the introduction and methods should be provided before concluding with this sentence.

Line 332: Table 3 section on surveillance and contact tracing -- ***Lassa Fever should be included***

***as a differential diagnosis for all pregnant women who die.***

What is the rationale for including ALL pregnant women? Including those dying from say eclampsia? With the limited Lassa fever testing, how would this be implemented?

Line 351: The data presented does not give evidence to the strong assertion on the outbreak attribution.

Discussion:

Include limitations in your current study that could affect interpretation of the findings
