# Supplementary material for: After action review of the response to an outbreak of Lassa fever in Sierra Leone, 2019: Best practices and lessons learnt
Source: PLoS Negl Trop Dis. 2022 Oct 5;16(10):e0010755. doi: 10.1371/journal.pntd.0010755 (PMC9534430; doi:10.1371/journal.pntd.0010755)
Supplement: Attachment — Submitted filename: Response to reviewers.docx [file pntd.0010755.s004.docx]

Fasil Tekola-Ayele

Associate Editor

PLOS Neglected Tropical Diseases

E-Mail: [em@editorialmanager.com](mailto:em@editorialmanager.com)

Dear Fasil Tekola-Ayele

**RE: PNTD-D-22-00190**

Thank you for your letter dated 22^nd^ April 2022 and the suggestions of the Editors and the two reviewers. We have carefully read through the peer review and have revised our paper accordingly.

Please find for your kind consideration the following:

1) A “point by point” response to the comments and suggestions of the reviewers
2) A new revised version of the manuscript. We have attached a highlighted copy using track changes and also attached a clean copy

Changes have also been made to improve reader clarity. We have also attached two supplementary materials as requested by the reviewers: the guidelines used to conduct the AAR and a reporting template.

Hoping these changes will meet your kind consideration. We are available for further information you might require.

Yours Sincerely,

Charles Njuguna

Author for correspondence

**POINT BY POINT RESPONSES TO THE REVIEWER COMMENTS**

**PNTD-D-22-00190**

**Responses to Comments by the Editors**

Many thanks for reviewing the manuscript and your insightful comments. We have revised the manuscript in line with your suggestions. We have provided a point-by-point response to your comments and suggestions. Your comments are highlighted in bold and our responses follow using italics.

**1) Adequate justification is needed why the lead AAR team members were part of the Ministry of Health, which was leading implementation. The impact of this on the reported findings and measures taken to mitigate this need to be adequately justified in the manuscript.**

***Response:*** *We have provided extra information that the AAR* *was guided* *by the country guidelines* ***‘****Learning from Incidents and Exercises: Guidance on EPRR Debriefing and After Action Reviews’ (Supplementary file 1). The document is a guide on how to conduct AARs and was adapted from the WHO guidelines for conducting AAR (see line 208-211 in the highlighted manuscript). According to these guidelines, the Lead facilitator is supposed to be independent while the support facilitators can be selected from government ministries, departments and agencies. We have therefore clarified this in line 226-231. To mitigate against bias, we have also clarified that team members of each pillar also had participants from other MDAs and partners who were not necessarily directly involved in the response (line 239-244). Clarification has also been made that the AAR was done in two phases and that the 2^nd^ phase comprised a bigger group that had multiple stakeholders that vetted the pillar level findings to ensure objectivity and impartiality (line 267-269)*

**2) Action taken after the AAR was not clear. AAR is not evaluation, so the follow up action is very important. It appears from the paper that the authors have developed an Action Plan at the end, but the content of the stated action plan is unclear.**

 ***Response:*** *We have clarified that a total of 60 recommendations were made during the AAR and the distribution per pillar has been indicated in the results section. We have also clarified that all pillars were requested to meet later and develop detailed action plans with timelines to implement the recommendations (Line 408-413). However, we were not able to get data on the status of the implementation of these recommendations/actions plans and this has been put as a limitation in the discussion section (line 507-512)*

-----------------------------------------------------------------------------------------------------------------------

**Responses to the Comments by Reviewer #1**

Many thanks for reviewing the manuscript and your insightful comments. We have revised the manuscript in line with your suggestions. We have provided a point-by-point response to your comments and suggestions. Your comments are highlighted in bold and our responses follow using italics.

**Methods**

**Although this article does not follow the classical structure of a Research paper (and how should it?), there is a very clear structure and the important aspects of the results are picked up in the discussion. Altogether, the Methods section could be shortened a bit to make it more readable.

As the objective was to cover the AAR, the design of the manuscript is adequate**

***Response:*** *We have re-structured the methods section to ensure that it follows the structure of a research paper. Specifically, we have introduced subheadings on Study setting and design and data collection and analysis (Line 201-276)*

**It would be informative to the Reader to have the template used for reporting available as supplementary material. A function of the paper would also be to guide future AARs with respect to the application in different Settings**.

***Response****: Both the country guidelines for conducting AAR (Learning from Incidents and Exercises: Guidance on EPRR Debriefing and After Action Reviews) (supplementary file 1) and the reporting template (supplementary file 2) have been provided as supplementary materials. The suggested function of AAR has been included in line 125.*


**Results**

**p. 12 first Paragraph. the authors found that it was failed to suspect LF as a differential in the index case with Fever and vaginal bleeding**. **While it is absolutely important to state that this presentation is at high Risk for LF, it is also true that HCW can fail to define the suspicion for a multitude of reasons. It would be important to know if there are any Guidelines or if there is any case Definition (for non-outbreak Situation probably) that could have helped make this differential diagnosis or anything that could guide early case detection in SL.**

***Response:*** *The original text referred to in page 12, Paragraph 1 was deleted as part of the document clean up. This comment has now been addressed under ‘Surveillance and Laboratory’ section. We have added information that surveillance for Lassa Fever in Sierra Leone is done under the case definition for Acute Viral Hemorrhagic Fever (AVHF) and if this case was picked as an AVHF then Lassa Fever would have been picked much earlier (Line 372-375).*

**It is stated in Table 3 that it should be included as a differential diagnosis in pregnant women who die, however it would be important to define how this Information can be delivered to HCW in a better Fashion.**

***Response:*** *The sentence in Table 2 has been revised and now reads as follows: ‘Lassa Fever should be included as a differential diagnosis for all pregnant women who die with fever due to the high maternal mortality in Sierra Leone. A blood sample should therefore be taken for testing before burial’ (Line 415)*

**P.14 l. 293. Which form of Ribavirin was used? p.o. / i.v.**

***Response:*** *Intravenous Ribavirin was used and this has been added (line 366)*

**Conclusions**

**Reviewer #1: Conclusions are very clearly presented. Limitations not so clearly. What could have been achieved more in the AAR?
Public Health relevance is clearly addressed.**

***Response****: Limitations have been added in the last paragraph of discussion section (line 507-512)*


**Editorial and Data Presentation Modifications?**

**Reviewer #1: The manuscript is written very clearly and concisely, and is by itself informative. It would be very helpful if the authors could share some of the Underlying documents 7 templates / Guidelines as suggested above and below.**

***Response:*** *The country AAR guidelines have been added as supplementary materials and it contains all the required templates used. Additionally, the reporting template has also been added separately as a supplementary material*


**Summary and General Comments**


**Minor comments:**
**- p. 10 last Paragraph. It would be interesting to Quote the source of the Country Guidelines or even offer them as supplementary material it this is allowed, to inform the Reader of the Framework of the AAR The same is true for p. 11 line 217 concerning the templates**

***Response****: The country guidelines for conducting AAR have been provided as supplementary material l (Supplementary file 1). The guidelines have all the templates but the pillar reporting template has also been provided separately as a supplementary material(Supplementary file 2)*

**The mortality is very high in this small cluster, compared to the mortality in endemic Areas. Maybe the authors could compare this to data published for endemic / non-endemic Areas? are there reasons to believe that mortality could be improved due to implementation of AAR results?**

***Response:*** *The requested extra information has been added in the discussion section line 427-438. The overall CFR was 60% in this cluster compared to an average of 39% CFR among all cases confirmed in Sierra Leone in 2016-2019 though higher at 63% among admitted patients in the same period. So yes we believe that challenges unearthed during AAR can help reduce this mortality if they are addressed.

---------------------------------------------------------------------------------------------------------------------------*

**Responses to the Comments by Reviewer #2**

Thank you for your review and insightful comments and suggestion on the manuscript. We have revised the manuscript in line with your suggestions. We have provided a point-by-point response to your comments and suggestions. Your comments are highlighted in bold and our responses follow using italics.

**Conclusions**

**Some of the conclusions not supported by the data provided. Limitations of the study have not been included**

***Response:*** *The Conclusions section has been edited to ensure that it is supported by available data (Line 516-530). Limitations have been added in the last paragraph of Discussion section (Line 507-512)*

**The manuscript is based on an after-action review (AAR) of a Lassa fever outbreak in Sierra Leone as a mechanism for improving on preparedness and response actions to public health outbreaks. The AAR is an important evaluation of disease outbreak response and documenting such findings could be useful in countries with similar contexts.**

**Major comment
My main concern is that the manuscript does not follow appropriately or provide the information expected in each section of a research article. It seems to be a written as a report which was then loosely attempted to be turned into a manuscript**.

***Response:*** *We have re-structured the methods section to ensure that it follows the structure of a research paper. Specifically, we have introduced subheadings on Study setting and design and data collection and analysis (Line 201-276)*

**Other comments**

**Abstract: The authors state that the outbreak was caused by a breach of IPC protocols. No evidence is provided in the results to back this assertion**.

***Response****: The evidence has now been provided in line 31-32*

**Line 66: It is not apparent to the reader that the simulation exercise and capacity assessments the authors refer to are among the four components of the IHR monitoring framework**

***Response****: To address the confusion, revision has been made to line 63-66 to show the 4 components of the IHR monitoring and evaluation framework (simulation, AAR, State Party Annual Report and Joint External Evaluation)*

**Line 68: Are AARs part of organizational learning? That needs to be clarified so that the paragraph can have coherence**

***Response****: AAR is part of organization learning and this has been clarified appropriately line (Line 72-73)*
 **Line 76: Use the appropriate citation style for the journal**

***Response****: The citation has been corrected (Line 81)*

**Line 92: Give a reference for the sentence**

***Response:*** *The reference has been added (Line 98)*

**Line 90 to 104: The two paragraphs are redundant and repetitive. The rationale for AARs is already stated in the introductory paragraph.**

***Response:*** *The two paragraphs have been deleted (line 98 to 110)*

**Line 116**: Incident not incidence

***Response:*** *The word ‘incidence’ has been replaced with ‘incident’ (Line 127***)**

**Line 121: Wasn’t the outbreak occurrence already known after the notification from Netherlands?** 

***Response:*** *The sentence has been edited to make it clear (Line 128-129)*

**Line 129 to 143: This description of the response should be in the results section.**

***Response:*** *The section (Coordination of the Outbreak Response) has been transferred from the introduction section to the Results section under ‘Coordination of the Response at the National and District Level’ (deleted from Line 140-155) Therefore, editing in the results section has been done to remove redundancy and repetition (Line 278-344)*

**Line 171:** **This subsection should be made brief and moved to before the incident description**

***Response:*** *The section has been moved to before the incident section (as part of introduction) and summarized into only two sentences (line 122-125)*

**Line 183: The table should be moved to methods**

***Response:*** *Table 1 has been moved to Methods section (Line 219)*

**Line 190-193:  Did the authors use the WHO guidance on AAR as well?**

***Response****: The paragraph has been edited to indicate that the country used its own guidelines for conducting AARs that have been adapted from the WHO guidelines (Line 208-211). The country guidelines have been attached as supplementary file 1.*

**Line 196: What were the considerations or qualities of the lead facilitator? Experience in AAR?**

***Response****: The line has been edited to indicate that the lead facilitator was an independent consultant hired by WHO and who had many years’ experience in conducting AARs (Line 226-231)*

**Line 204: What did the presentation template for the pillar discussions contain? Alternatively include it as a supplementary document.**

***Response:*** *Information on what the presentation template contained has been added (Line 245-247) and the template has also been added as supplementary file 2*

**Line 206: Give a reference for the document. Were the participants trained on conducting AAR besides being provided with the document.**

***Response****: The document has been added as a supplementary file 1. The line has also been edited to indicate the participants were trained on the document (Line 248)*

**Line 229: The coordination description is already stated in the introduction and methods should be provided before concluding with this sentence.**

*Response: This concluding sentence has been shifted from the first paragraph under coordination to the latter part of the section after adequate description of the response coordination has been done (Line 296-297)*

**Line 332: Table 3 section on surveillance and contact tracing --  Lassa Fever should be included as a differential diagnosis for all pregnant women who die**.
   **What is the rationale for including ALL pregnant women? Including those dying from say eclampsia? With the limited Lassa fever testing, how would this be implemented?**

***Response****: The sentence in Table 3 has been edited to include fever. Sierra Leone has the highest maternal mortality in the world so this has also been added. The sentence now reads as follows ‘Lassa Fever should be included as a differential diagnosis for all pregnant women who die with fever due to the high maternal mortality in Sierra Leone’* (Line 415)

**Line 351: The data presented does not give evidence to the strong assertion on the outbreak attribution.**

***Response:*** *The sentence has been edited to provide the evidence. The sentence now reads as follows: “This outbreak was attributed to a breach in IPC protocols due to the fact that all four secondary cases were related to a single surgical operation” (Line 427-428)*

**Discussion:**
Include limitations in your current study that could affect interpretation of the findings

***Response:*** *Limitations have been added in the last paragraph of discussion (Line 507-512)*
