# Supplementary material for: After action review of the response to an outbreak of Lassa fever in Sierra Leone, 2019: Best practices and lessons learnt
Source: PLoS Negl Trop Dis. 2022 Oct 5;16(10):e0010755. doi: 10.1371/journal.pntd.0010755 (PMC9534430; doi:10.1371/journal.pntd.0010755)
Supplement: Attachment — Submitted filename: Response to reviewers.docx [file pntd.0010755.s005.docx]

Fasil Tekola-Ayele
Academic Editor
PLOS Neglected Tropical Diseases

Andrea Marzi
Section Editor
PLOS Neglected Tropical Diseases

Dear Fasil and Andrea

**RE: PNTD-D-22-00190R1**

Thank you for your email and the suggestions of the reviewers on the revised manuscript. We have carefully read through the suggestions and have revised our paper accordingly.

Please find for your kind consideration the following:

1) A “point by point” response to the comments and suggestions of the reviewers
2) A new revised version of the manuscript. We have attached a highlighted copy using track changes and also attached a clean copy

We have also addressed the additional instructions as follows:

- Figure files: There is only one figure (Fig 1) and it has been converted into Fig1.tif using the PACE tool
- Data requirements: All data used is contained within the manuscript and this has been indicated in the manuscript
- Reproducibility: We do not have any laboratory protocols in this manuscript
- References: These have been counterchecked. One reference (previously #20) has been deleted and so the references are now 21 and not 22

In addition to the above changes, we have also formatted the headings and sub headings as per journal instructions.

Hoping these changes will meet your kind consideration. We are available for further information you might require.

Yours Sincerely,

Charles Njuguna

Author for correspondence

**POINT BY POINT RESPONSES TO THE REVIEWER COMMENTS**

PNTD-D-22-00190R1

**Responses to the Comments by Reviewer #1**

No comments from Reviewer 1 were seen

**Responses to the Comments by Reviewer #2**

Thank you for your review and insightful comments and suggestion on the manuscript. We have revised the manuscript in line with your suggestions. We have provided a point-by-point response to your comments and suggestions. Your comments are highlighted in bold and our responses follow using italics.

**The manuscript has improved from the previous versions and the reviewer comments have mostly been addressed.
See below minor comments. In lieu of absent line numbers, i have tried to be as descriptive as possible on the referenced lines or sentences.**

*Response: Line numbers have now been added for ease of reference (we apologize for this as we are not sure how they disappeared in the previous submission)*

**Abstract page 2, results, first sentence: Causation on an IPC breach resulting in transmission of Lassa fever was not established based on the presented results. Use terms such as associated, likely occurred. This comment applies to the other sections of the manuscript where similar assertions are made. It is important not to use language that imply causation without the requisite data or evidence.**

*Response: The suggestion has been fully taken care of and corrections have been made by using then term ‘likely occurred’ in all relevant areas (Abstract section, line 32; Author summary section, line 53; Discussion section, line 331)*

**Introduction section, page 4, first paragraph: The manuscript is lengthy and removing this paragraph would help because the paragraph is unnecessary because the aim of AAR is already well stated in the previous paragraph**

*Response: The suggested paragraph has been deleted(Line 70-76)*

**Introduction section, page 6, 3rd last line: Did this patient have a febrile illness at the time of admission or surgery? This will help the reader appreciate if the patient could have already been infected.**

*Response: Yes, the patient developed fever 14 days after the surgical operation and then died 3 days later. This has been added in the manuscript, line 136-137*

**Methods, page 7 second last line: The MoHS guidance included in the supplementary provides that the AARs are within 6 weeks of the incident. Clarify why it is inferred in the current text that AARs are expected to be conducted within three months. Is it in reference to a guidance different from that from MoHS.**

*Response: Clarification has been made that the 3 months is in reference to WHO AAR guidelines which is the maximum recommended period (Line 163-164). We used WHO reference as that is the reference globally*

**Page 12, case management and IPC section, first line:  Was there specific information of IPC breaches during the surgery on the index case? Is it that the surgical team did not use gloves, surgical gowns or masks?**

*Response: From the information that was collected during the investigations, it was clear from other hospital staff that there were several incidences where the patient (index case) was examined or handled without use of gloves. However, we have deliberately omitted this information as we do not have concrete evidence on it as the affected staff did not out rightly reveal this information. This is why we have opted to say ‘potential breach in IPC guidelines’ (Line 250)*

**Page 16, Discussion first two sentence: Remove sentence section after the comma because it is redundant.**

*Respons*e: *The section has been deleted as recommended(Line 322-323)*

**Page 17, discussion, first paragraph, line 8 and 9: rewrite the cases by year for ease of readability.  .. “15 to 35 cases annually between 2016-2019”**

*Response: The cases have been rewritten as advised and now the sentence reads as follows*: ‘*The total number of laboratory confirmed Lassa Fever cases reported in Sierra Leone was 15 to 35 cases annually from 2016 to 2019’ (Line 338-339)*

**Page 20, second last paragraph on limitations:  An important limitation to include in this study is that the some of the data was based on self-reports that could be biased especially among the staff who may have been expected to take “best practice” actions but did not. There might have been fear of "self-incrimination". The bias could have been reduced by triangulating the information with reports and documentation during the outbreak and before the AAR.**

*Response: The suggested limitation on possible bias has been added in the limitation section (Line 409-412)*
